# Political Honors to Physicians

**Published:** 1874-10

**Authors:** 


*Political Honors to Physicians.*

It is with very great pleasure we see so many good and able medical gentlemen of the State sent, by appreciative constituents, to the General Assembly of Georgia.

Dr. W. O. Daniel, a noble brother and an honest, warm-hearted man, takes a seat in the Senate. The interests of the people and profession are safe in the hands of our esteemed friend, and we doubt not he will make a "splendid" record as a Senator.

Dr. J. G. Thomas, of Savannah, goes to the Lower House. Dr. Thomas is a professor in the Savannah Medical College; and, being a gentleman of culture and talent, fills every place thrust upon him with ability. We congratulate our friend upon his promotion. The Georgia Medical Society will find an able and earearnest advocate in Dr. Thomas.

Dr. H. H. Carleton, of Athens, the political war-horse of the profession, has been returned to his old place in the House. He does good service whenever he works.

Dr. T. H. Baker, of Bartow—our old Bartow—heads his ticket in the "empire" county, and for the second time takes his place in the House. The Doctor has made a fine reputation as a legislator, and will wield a powerful influence for the interests of the profession.

We congratulate these brethren and the profession no less upon their elevation to position. That they will do honor to both, we do not question. W. T. G.

